# ASO Author Reflections: Smaller Tumors and Negative Nodes—Current Selection to Surgery in Multiple Intrahepatic Cholangiocarcinoma. A Future Role for Oncological Therapies to Improve Outcomes and Permit Resection for More Patients?

**DOI:** 10.1245/s10434-026-19117-y

**Published:** 2026-01-20

**Authors:** Hannes Jansson, Helena Taflin, Bergthor Björnsson, Jozef Urdzik, Oskar Hemmingsson, Jenny Lundmark Rystedt, Stefan Gilg, Per Sandström, Ernesto Sparrelid

**Affiliations:** 1 Division of Surgery and Oncology, Department of Clinical Science, Intervention and Technology, Karolinska Institutet, Karolinska University Hospital, Stockholm, Sweden; 2 Department of Transplantation, Institute of Clinical Sciences, Sahlgrenska Academy, University of Gothenburg, Gothenburg, Sweden; 3 Department of Transplantation at Sahlgrenska University Hospital, Region Västra Götaland, Gothenburg, Sweden; 4 Department of Biomedical and Clinical sciences, Linköping University, Linköping, Sweden; 5 Clinical Department of Surgery in Linköping, Region Östergötland, Linköping, Sweden; 6 Department of Surgical Sciences, Uppsala University, Uppsala, Sweden; 7 Department of Diagnostics and Intervention, Umeå University, Umeå, Sweden; 8 Department of Surgery, Skåne University Hospital, Lund, Sweden; 9 Department of Clinical Sciences, Lund University, Lund, Sweden

## Past

A significant proportion of patients diagnosed with intrahepatic cholangiocarcinoma (iCCA) present with localized disease but multiple hepatic lesions.^1,2^ While tumor multiplicity has been assessed as a contraindication to surgery,^3^ updated guidelines assert that resection can be considered in selected patients.^4^ However, the level of available evidence is low, with only limited data on outcomes after resection for multiple iCCA.^4^

## Present

In a national population-based analysis, outcomes were assessed for all patients undergoing surgery for iCCA during the period 2010–2021.^5^ Patients undergoing resection for iCCA with two or three lesions had a median overall survival (OS) of 27.1 months (95% confidence interval [CI] 18.8–35.5). This outcome was dependent on selection, as patients with resection had better performance status (*P* = 0.007), smaller tumors (*P* = 0.004), and more often negative lymph nodes (*P* = 0.028) as compared with patients with localized multiple iCCA and nonresection.

While patients with two or three lesions achieved better outcomes with resection than after exploration (*P* < 0.001), patients with four or more lesions did not have better outcomes with resection (*P* = 0.922). When controlling for tumor number and the abovementioned selection factors in a multivariable analysis, resection was not significantly associated with OS (*P* = 0.090). This illustrates how long-term outcomes after resection for multiple iCCA depend on adequate selection, and that a combination with other high-risk factors such as larger size, lymph node metastasis, and poor patient condition will likely negate any benefit from surgery. Importantly, in the current cohort, most of the study period preceded the introduction of adjuvant capecitabine as standard therapy, and only less than 40% of patients received adjuvant treatment. Preoperative chemotherapy was only used for 6.5% of patients with multiple iCCA. The rate of recurrence within 6 months was 40% for patients with two or three lesions and 75% for patients with four or more lesions.

## Future

While these results support consideration of resection as one option for patients with a limited number of lesions, smaller tumors, good performance status, and no clinical signs of lymph node metastasis, an elevated risk of early recurrence motivates further study of targeted and neoadjuvant systemic and locoregional oncological approaches in multiple iCCA. Future studies also need to assess whether such oncological strategies can permit resection and improve long-term outcomes for patients with a higher number of lesions or other concurrent high-risk factors.
